# Study protocol of the C-SOLVE (JACCRO GC-12) trial; a randomized, non-comparative phase II trial of CapeOX or SOX plus zolbetuximab in HER2-negative, CLDN18.2-positive unresectable advanced gastric or esophagogastric junction cancer

**DOI:** 10.1186/s12885-026-16478-1

**Published:** 2026-07-17

**Authors:** Satoshi Yuki, Hisato Kawakami, Kazuaki Harada, Yohei Kubota, Kyongsun Pak, Yoshihiro Kakeji, Masanori Terashima, Eishi Baba, Kei Muro, Yu Sunakawa, Wataru Ichikawa, Masashi Fujii

**Affiliations:** 1 Department of Cancer Genomics and Precision Medicine, Hirosaki University Graduate School of Medicine, Hirosaki, Japan; 2 Department of Clinical Oncology, Tohoku University Graduate School of Medicine, 4-1 Seiryo-machi, Aoba-ku, Sendai, 980-8575 Miyagi Japan; 3 Divison of Cancer Center, Hokkaido University Hospital, Sapporo, Japan; 4 Department of Clinical Oncology, St. Marianna University School of Medicine, Kawasaki, Japan; 5 Division of Biostatistics, Department of Data Management, Center for Clinical Research, National Center for Child Health and Development, Tokyo, Japan; 6 Division of Gastro-intestinal Surgery, Department of Surgery, Kobe University Graduate School of Medicine, Kobe, Japan; 7 Division of Gastric Surgery, Shizuoka Cancer Center, Shizuoka, Japan; 8 Department of Comprehensive Oncology, Graduate School of Medical Sciences, Kyushu University, Fukuoka, Japan; 9 Department of Clinical Oncology, Aichi Cancer Center Hospital, Nagoya, Japan; 10 Department of Medical Oncology, Sonoda Daiichi Hospital, Tokyo, Japan; 11 Japan Clinical Cancer Research Organization (JACCRO), Tokyo, Japan

**Keywords:** CLDN18.2-positive, Rirst-line chemotherapy, Oxaliplatin, S-1, Zolbetuximab

## Abstract

**Background:**

Capecitabine + oxaliplatin (CapeOX) + zolbetuximab and 5-FU + oxaliplatin (mFOLFOX6) + zolbetuximab are recommended as first-line treatments for chemotherapy-naïve patients with HER2-negative, CLDN18.2-positive advanced gastric or esophagogastric junction cancer. However, no prospective trials have evaluated the efficacy and safety of S-1 + oxaliplatin (SOX) + zolbetuximab. This study is planned as a non-comparative, prospective, randomized phase II clinical trial to evaluate the efficacy and safety of SOX + zolbetuximab in chemotherapy-naïve patients with HER2-negative, CLDN18.2-positive advanced gastric or esophagogastric junction cancer.

**Methods/design:**

This randomized study will enroll patients ≥ 18 years old with histologically confirmed HER2-negative, CLDN18.2-positive (IHC defined as ≥ 75% of tumour cells showing moderate-to-strong membranous CLDN18 staining) advanced gastric or esophagogastric junction cancer. Participants (*n* = 140) will be randomly assigned (1:1) to receive either SOX + zolbetuximab or CapeOX + zolbetuximab. The primary endpoint is the 12-month progression-free survival (PFS) rate, with a threshold of 17% and an expected rate of 33% for SOX + zolbetuximab. To account for a 10% deviation risk, the target enrollment for each arm is 70 patients. CapeOX + zolbetuximab will serve as a prospective observational arm without hypothesis testing. Secondary endpoints will include PFS, overall survival, objective response rate, disease control rate, duration of response, time to response, and safety. The enrollment period is 2 years, followed by a 2-year observation period.

**Discussion:**

Since replacing capecitabine with S-1 should provide the same level of therapeutic efficacy, SOX + zolbetuximab may be increasingly used to treat advanced gastric or esophagogastric junction cancer in Asian countries. Verifying the efficacy and safety of SOX + zolbetuximab is thus an important task.

**Trial registration:**

The protocol has been registered at the website of the Japan Registry of Clinical Trials (JRCT), Japan (protocol ID jRCTs031240510, protocol version: 1.11) on November 26, 2024. The details are available at https://jrct.mhlw.go.jp/.

**Supplementary Information:**

The online version contains supplementary material available at https://doi.org/10.1186/s12885-026-16478-1.

## Background

Gastric cancer is the sixth most common malignancy and the third leading cause of cancer-related mortality worldwide, with more than 60% of cases occurring in East Asia, including Japan, Korea, and China [1]. In recent years, the combination of immune checkpoint inhibitors with platinum and fluoropyrimidine chemotherapy has become an internationally recognized standard first-line treatment for patients with HER2-negative unresectable or recurrent gastric and gastroesophageal junction (GEJ) cancer, based on the results of several global phase III trials [2–5].

In Japan, the development of systemic chemotherapy has historically centered on an oral fluoropyrimidine, S-1. The JCOG9912 trial demonstrated the non-inferiority of S-1 monotherapy compared with continuous infusion of 5-fluorouracil (5-FU) [6]. The SPIRITS trial subsequently showed the superiority of S-1 plus cisplatin (SP) over S-1 monotherapy in terms of overall survival (OS) [7]. Further, the G-SOX trial confirmed the non-inferiority of S-1 plus oxaliplatin (SOX) compared with SP, leading to the recognition of SOX as a standard first-line regimen for unresectable or recurrent gastric cancer in Japan [8]. At present, SOX has also been adopted as a backbone regimen in combination with targeted agents such as trastuzumab and immune checkpoint inhibitors, underscoring its central role in the treatment of gastric cancer in Japan. Real-world treatment patterns in Japan confirm the predominance of S-1-based regimens. In the G-KNIGHT study, which evaluated nivolumab plus chemotherapy in routine clinical practice, 74.6% of patients received SOX, whereas CapeOX and FOLFOX accounted for far fewer cases [9]. These data underscore the entrenched role of S-1 in Japan, in contrast with international trials that have typically employed capecitabine- or 5-FU-based regimens.

Recently, the anti-CLDN18.2 monoclonal antibody zolbetuximab (ZOL) has been established as the first-in-class therapy targeting this tight junction protein [10]. In the pivotal phase III SPOTLIGHT trial, the addition of ZOL to mFOLFOX6 significantly improved both progression-free survival (PFS) and OS compared with placebo [11]. Similarly, the GLOW trial demonstrated significant survival benefit with ZOL in combination with CapeOX compared with CapeOX alone [12]. Across both studies, gastrointestinal toxicities such as nausea and vomiting were more frequent with ZOL but were considered clinically manageable. Based on these results, ZOL was approved in Japan in 2024, and CapeOX + ZOL and FOLFOX + ZOL are now recommended regimens in the 2025 Gastric Cancer Treatment Guideline [13].

However, in Japanese clinical practice, FOLFOX is used only in limited circumstances, and SOX is generally preferred when an oral fluoropyrimidine is selected. Importantly, S-1 has demonstrated particular efficacy against poorly differentiated gastric cancer, appearing to provide greater benefit than capecitabine [14]. Given that CLDN18.2-positive tumors are frequently poorly differentiated, SOX may represent a promising partner regimen for ZOL in Japan. However, prospective evidence supporting this combination is currently lacking. Despite this strong clinical rationale, no prospective data are currently available. We therefore designed this randomized, non-comparative phase II trial to evaluate the efficacy and safety of SOX + ZOL, using CapeOX + ZOL as a reference, in patients with HER2-negative, CLDN18.2-positive unresectable or recurrent gastric/GEJ cancer.

## Methods/design

### Study objective

This protocol follows the SPIRIT 2025 guidelines, and the completed SPIRIT checklist is provided as Supplementary Material 1.

This trial is aimed at exploring and evaluating the safety and efficacy of SOX + ZOL as a first-line treatment for HER2-negative, CLDN18.2-positive advanced gastric or esophagogastric junction cancer. The primary endpoint is the 12-month PFS rate by investigator’s assessment. The secondary endpoints are PFS, OS, objective response rate (ORR), disease control rate, duration of response, time to response, and safety. Adverse events will be graded according to the Common Terminology Criteria for Adverse Events (Japanese edition, Japan Clinical Oncology Group (JCOG) version v5.0).

### Study design

**Fig. 1:**
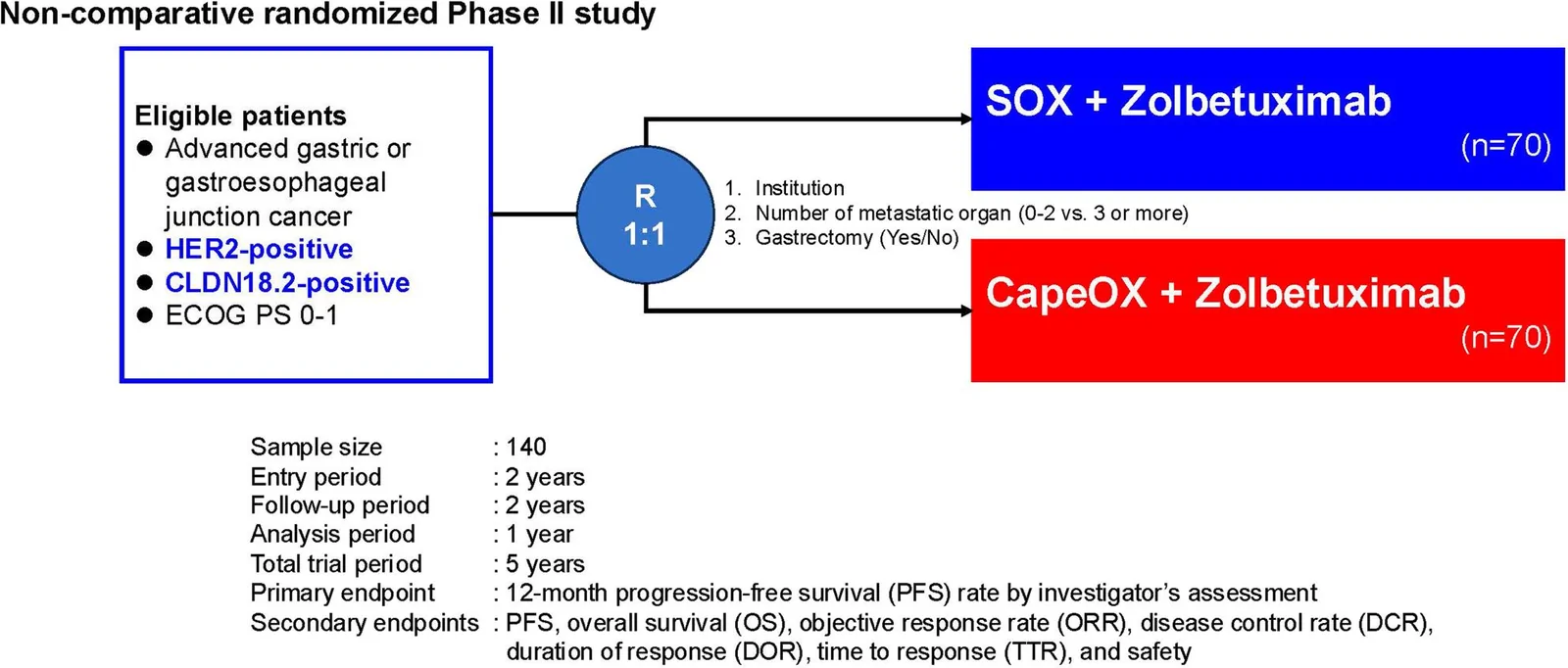
C-SOLVE_Protocol_Paper_Study schema

The C-SOLVE (JACCRO GC-12) trial is an open-label, prospective, multicenter randomized, non-comparative phase II trial that will be conducted at 112 centers of the Japan Clinical Cancer Research Organization (JACCRO) in Japan (Fig. 1).

After written informed consent has been obtained, eligible patients will be randomized (1:1) to either the SOX plus ZOL or the CapeOX plus ZOL. Stratification factors will include: (1) institution; (2) number of metastatic sites (0–2 vs. >3); and (3) prior gastrectomy (yes vs. no). A researcher performs the randomization procedure using a secure online randomization system.

This trial will be conducted in accordance with the World Medical Association Declaration of Helsinki and Japanese Ethical Guidelines for Medical and Health Research Involving Human Subjects. The trial protocol has been approved by Nihon University Itabashi Hospital Certified Clinical Research Review Board (CRB3180013). The protocol of this study has been registered in the Japan Registry of Clinical Trials (JRCT) (jRCTs031240510; November 26, 2024). Any important modifications to the trial protocol required approval from the Nihon University Itabashi Hospital Certified Clinical Research Review Board. Approved amendments were updated on JRCT (jRCTs031240510) and communicated to the research team and, where applicable, to enrolled participants. 

### Inclusion criteria

To be eligible for this trial, patients must meet the following criteria:

1. Histologically confirmed adenocarcinoma of the gastric or gastroesophageal junction
2. Eastern Cooperative Oncology Group performance status (ECOG PS) of 0 or 1
3. ≥18 years old at the time of providing consent
4. Have undergone HER2 testing and have been confirmed as HER2-negative
5. Have undergone CLDN testing and have been confirmed as CLDN18.2-positive
6. At least one evaluable lesion (measurable and/or non-measurable) according to RECIST version 1.1, based on imaging assessment within 28 days prior to enrollment.
7. Capable of oral intake
8. Unresectable advanced or recurrent gastric cancer with no prior history of chemo- or radiotherapy for the disease.
9. Meeting the following criteria for major organ function within 7 days prior to enrollment (the same day of the previous week is acceptable):
  1. Hemoglobin: ≥9.0 g/dL without a transfusion within 14 days prior to enrollment.
  2. Neutrophil count: ≥1,500 /mm³.
  3. Platelet count: ≥100,000 /mm³ (10.0 × 10⁴/mm³).
  4. Total bilirubin: ≤2.0 mg/dL.
  5. Aspartate aminotransferase (AST): ≤100 U/L (≤ 150 U/L for patients with liver metastases).
  6. Alanine aminotransferase (ALT): ≤100 U/L (≤ 150 U/L for patients with liver metastases).
  7. Creatinine clearance (CCr): ≥50 mL/min. Estimated CCr should be ≥ 50 mL/min using the Cockcroft–Gault equation. If the estimated value is < 50 mL/min, the patient is eligible if the measured value using 24-h pooled urine is confirmed to be ≥ 50 mL/min. For patients assigned to the SOX + ZOL group with a CCr of 50–60 mL/min, S-1 should be initiated at a reduced dose level.
10. Written informed consent from the patient.

### Exclusion criteria

Patients who meet any of the following criteria are not eligible for the study:

1. History of prior treatment with CLDN18.2-targeted therapy
2. History of hypersensitivity to S-1, capecitabine, fluorouracil, oxaliplatin, or other platinum-based agents
3. Recurrence within 180 days after completing preoperative adjuvant chemotherapy or chemoradiotherapy that included platinum-based agents
4. Recurrence during or within 180 days after completing postoperative adjuvant chemotherapy that included fluoropyrimidine-based agents
5. Known presence of microsatellite instability-high (MSI-H) or mismatch repair deficiency (dMMR)
6. Surgical treatment within 14 days prior to enrollment
7. Synchronous multiple primary cancers or metachronous multiple primary cancers within a disease-free interval of five years. However, epithelial carcinomas or mucosal carcinomas considered curable by local treatment are not regarded as active multiple primary cancers.
8. Symptomatic metastases to the central nervous system (brain, spinal cord, or meninges)
9. Grade 2 or higher nausea, vomiting, or peripheral sensory neuropathy
10. Infection requiring systemic treatment
11. Uncontrolled diabetes mellitus despite continuous insulin use
12. Psychiatric disorders or symptoms deemed to interfere with study participation
13. Suspicion of dementia or cognitive impairment, scoring ≤ 2 on the Mini-Cog test
14. Pregnant women, lactating women who wish to continue breastfeeding, or patients who refuse contraception
15. Patients deemed inappropriate for study participation by the investigator

### Study treatment

Patients will be randomly assigned to receive one of the following treatments:

SOX plus ZOL arm: intravenous infusion of ZOL at 800 mg/m^2^ (cycle 1, day 1) followed by 600 mg/m^2^ (day 1 of subsequent cycles) plus SOX (oral S-1 at 40 mg/m^2^, twice daily on days 1–14 of each cycle; intravenous infusion of oxaliplatin at 130 mg/m^2^, day 1 of each cycle) repeated every 3 weeks until eight cycles.

CapeOX plus ZOL arm: intravenous infusion of ZOL at 800 mg/m^2^ (cycle 1, day 1) followed by 600 mg/m^2^ (day 1 of subsequent cycles) plus CapeOX (oral capecitabine at 1000 mg/m^2^, twice daily on days 1–14 of each cycle; intravenous infusion of oxaliplatin at 130 mg/m^2^, day 1 of each cycle) repeated every 3 weeks until eight cycles. Patients continued beyond eight cycles with ZOL plus S-1 or capecitabine, until disease progression, unacceptable toxicity, start of another anti-cancer treatment or other discontinuation criteria were met, as specified in the protocol.

In both arms, protocol treatment will be administered only after confirming that the neutrophil count is ≥ 1500/mm^3^, platelet count is ≥ 100,000/mm^3^, serum total bilirubin is ≤ 1.5 mg/dL, AST/ALT is ≤ 100 U/L (or ≤ 150 U/L in cases with liver metastases), creatinine clearance is ≥ 50 mL/min, and nausea/vomit/peripheral sensory neuropathy is grade 1 or lower.

The next cycle should not be administered unless the neutrophil count is ≥ 1000/mm^3^, platelet count is ≥ 75,000/mm^3^, serum total bilirubin ≤ 2.5 mg/ dL, AST/ALT ≤ 150 U/L (or ≤ 200 U/L in cases with liver metastases), creatinine clearance is ≥ 30 mL/min, anorexia/nausea/vomit is grade 1 or lower, and diarrhea is grade 1 or lower for SOX and CapeOX. However, ZOL may be administered as long as anorexia/nausea/vomiting is grade 1 or lower.

If the next cycle of the protocol treatment cannot be initiated within 28 days from the scheduled start date, protocol treatment will be discontinued. For oxaliplatin, S-1, and capecitabine, if adverse events requiring dose reduction occur prior to the start of a cycle, up to two dose reductions may be performed as needed. If more than two dose reductions are required, treatment with that drug must be discontinued. Once a dose reduction has been implemented, dose re-escalation in subsequent cycles is not permitted. In contrast, dose reduction is not allowed for ZOL.

### Concomitant care

No anticancer treatment other than the study treatment specified in this protocol is permitted during the protocol-defined treatment period. Specifically, chemotherapy other than S-1, capecitabine, and oxaliplatin; immunotherapy; antibody therapy other than zolbetuximab; radiotherapy; surgical treatment; and any other therapy that may interfere with the assessment of the study endpoints are prohibited. In addition, concomitant use of any investigational product, including those not intended for the treatment of cancer, is prohibited.

### Follow-up

Lesions will be measured with contrast-enhanced CT of the trunk every 9 weeks. All subjects will continue to undergo imaging unless disease progression is noted.

### Sample size and follow-up period

The planned enrollment target will be 70 patients in each arm. The duration of patient recruitment will be 2 years, the follow-up period will be 2 years from final patient enrollment, and the analysis period will be 1 year. The total duration of this trial will be 5 years. No formal interim efficacy analyses are planned for this trial.

### Statistical considerations

In previous reports [11, 12], combination therapy with ZOL did not demonstrate a sufficiently improved response rate compared to chemotherapy plus placebo. The primary endpoint of this study is therefore set as the 12-month PFS rate as assessed by the investigator. In the GLOW study, the investigator-assessed 12-month PFS rate was reported as 29.17% in the CapeOX plus ZOL group and 17.19% in the CapeOX plus placebo group in the overall population, and 56.81% and 13.40%, respectively, in the Japanese subpopulation [15].

Based on these results, the threshold and expected 12-month PFS rates for the SOX plus zolbetuximab regimen were estimated to be 17% and 33%, respectively. Assuming a one-sided alpha error of 2.5% and a beta error of 20%, the required sample size is calculated to be 63 patients. Taking into account an estimated dropout rate of approximately 10%, the target enrollment is set at 70 patients.

In addition, the CapeOX plus ZOL regimen will be evaluated in a prospective observational study without formal hypothesis testing, with a target enrollment of 70 patients, equal to that of the SOX plus ZOL group.

Statistical analyses will be conducted using intention-to-treat principles for efficacy data, with missing data handled using multiple imputation by chained equations (MICE) with predictive mean matching for continuous variables.

### Dissemination policy

The results of the C-SOLVE trial will be disseminated regardless of the direction or magnitude of findings. Primary outcomes will be submitted for publication in peer-reviewed journals in the fields of medical oncology, with secondary outcomes reported in subsequent publications. Findings will also be presented at relevant national and international scientific conferences. A summary of results will be posted on Japan Registry of Clinical Trials (jRCTs031240510) within 12 months of trial completion.

### Monitoring

The Data and Safety Monitoring Committee of the JACCRO will independently review protocol compliance, safety of the study, and the accuracy of data collection. This monitoring will be performed annually.

### Participating institutions

Aichi Cancer Center, Aichi Medical University Hospital, Akita University Hospital, Asahikawa Medical University Hospital, Cancer Institute Hospital, Chiba Cancer Center, Chiba University Hospital, Funabashi Municipal Medical Center, Gifu Prefectural General Medical Center, Gifu University Hospital, Gunma University Hospital, Hakodate Goryoukaku Hospital, Hakodate Municipal Hospital, Higashiosaka City Medical Center, Hiroshima City Hiroshima Citizens Hospital, Hiroshima City North Medical Center Asa Citizens Hospital, Hiroshima Prefectural Hospital, Hiroshima University Hospital, Hokkaido Cancer Center, Hokkaido University Hospital, Hyogo Prefectural Nishinomiya Hospital, Ibaraki Prefectural Central Hospital, Ichinomiyanishi Hospital, Institute of Science Tokyo Hospital, Ishikawa Prefectural Central Hospital, Iwamizawa Municipal General Hospital, JA Hiroshima General Hospital, Japanese Red Cross Akita Hospital, Japanese Red Cross Kitami Hospital, Japanese Red Cross Tokushima Hospital, JCHO Kyushu Hospital, Jisenkai Aizawa Hospital, Kagawa University Hospital, Kagoshima University Hospital, Kaizuka City Hospital, Kanagawa Cancer Center, Kanazawa Medical University Hospital, Kansai Medical University hospital, Kansai Rosai Hospital, Kawasaki Medical School Hospital, Kindai University Hospital, Kobe University Hospital, Kochi Health Sciences Center, Kochi Medical School Hospital, Komaki City Hospital, Kurashiki Central Hospital, Kurume University Hospital, Kushiro Rosai Hospital, Kyoto Katsura Hospital, Kyushu University Hospital, Matsuyama Red Cross Hospital, Minoh City Hospital, Nagano Municipal Hospital, Nagaoka Chuo General Hospital, Nagasaki Harbor Medical Center, Nagoya Medical Center, Nagoya University Hospital, Nakadori General Hospital, Nara Medical University Hospital, National Hospital Organization Fukuyama Medical Center, National Hospital Organization Kure Medical Center and Chugoku Cancer Center, National Hospital Organization Kyushu Medical Center, National Hospital Organization Osaka Medical Center, Nihon University Hospital, Nihon University Itabashi Hospital, Niigata Cancer Center Hospital, Niigata University Medical & Dental Hospital, Okayama University Hospital, Osaka General Medical Center, Osaka International Cancer Institute, Osaka Keisatsu Hospital, Osaka Medical and Pharmaceutical University Hospital, Osaka Rosai Hospital, Osaki Citizen Hospital, Rakuwakai Otowa Hospital, Rinku General Medical Center, Saga-ken Medical Center Koseikan, Saga University Hospital, Saiseikai Kumamoto Hospital, Saiseikai Niigata Hospital, Saiseikai Utsunomiya Hospital, Saiseikai Yokohama-shi Nanbu Hospital, Saitama Prefectural Cancer Center, Sakai City Medical Center, Sano Hospital, Sapporo City General Hospital, Seirei Mikatahara General Hospital, Sendai Kousei Hospital, Sendai Open Hospital, Shiga University of Medical Science Hospital, Shimane Prefectural Central Hospital, Shizuoka Cancer Center, Showa Medical University Hospital, Showa Medical University Fujigaoka Hospital, Showa Medical University Koto Toyosu Hospital, St. Marianna University Hospital, Suita Municipal Hospital, Teine Keijinkai Hospital, The Jikei University Hospital, The Jikei University Kashiwa Hospital, Tohoku University Hospital, Tokai University Hospital, Tokuyama Central Hospital, Tokyo Metropolitan Cancer and Infectious Center Komagome Hospital, Tottori University Hospital, Toyama Prefectural Central Hospital, Toyama University Hospital, Toyonaka Municipal Hospital, Wakayama Medical University Hospital, Yokohama City University Medical Center, and Yokohama Municipal Citizen’s Hospital.

## Discussion

The present trial is designed to address the clinical need for an evidence-based evaluation of SOX in combination with ZOL, given that S-1-based regimens are widely utilized in Japan. Although both CapeOX + ZOL and FOLFOX + ZOL are currently recommended based on the SPOTLIGHT [11] and GLOW [12] trials, these regimens do not reflect the predominant practice patterns in Japan, where SOX has become the standard oral fluoropyrimidine-platinum backbone.

The choice of SOX as the investigational partner regimen for ZOL is supported by both clinical practice patterns and biological rationale. S-1 has demonstrated superior efficacy in poorly differentiated gastric cancer, a histological subtype in which CLDN18.2 positivity is frequently observed [14]. This suggests a potentially synergistic benefit when combining SOX with ZOL in this molecularly and histologically defined subgroup. Moreover, SOX has practical advantages, including outpatient convenience and avoidance of continuous infusion devices, further reinforcing its suitability as a backbone regimen in Japan. In contrast, the CapeOX and FOLFOX combined regimen, although validated in international trials [2, 3], is less frequently utilized in Japan. The G-KNIGHT study confirmed that the vast majority of patients receiving nivolumab plus chemotherapy were treated with SOX [9], underscoring the clinical relevance of investigating SOX + ZOL.

ZOL is characterized by gastrointestinal toxicities such as nausea and vomiting [11, 12]. S-1 is associated with mucosal toxicities including diarrhea and stomatitis, whereas capecitabine is prone to cause hand-foot syndrome. Given these differing toxicity profiles, the potential for overlapping adverse events with SOX + ZOL makes prospective evaluation of safety particularly important. Moreover, now that ZOL has become widely available in clinical practice, effective toxicity management is a key determinant of its optimal use. This trial will provide important insights into how such toxicities are managed in real-world Japanese patients, thereby informing the safe and effective integration of SOX + ZOL into routine practice.

Although the pivotal SPOTLIGHT and GLOW trials demonstrated significant improvements in OS and PFS with the addition of ZOL to chemotherapy [11, 12], they did not show any clear additive benefit in terms of ORR. This finding highlights a limitation in using ORR as a surrogate marker of ZOL efficacy. Consequently, a conventional single-arm phase II trial with ORR as the primary endpoint was deemed inappropriate. Instead, times to event endpoints such as PFS are more suitable. However, PFS is highly influenced by patient background, making the interpretation of results difficult in a single-arm setting. For this reason, a randomized, non-comparative phase II design has been adopted, with CapeOX + ZOL serving as the reference. This approach is particularly important in light of the unexpectedly favorable PFS observed in Japanese subgroups of the SPOTLIGHT and GLOW trials, where outcomes may have been influenced by factors such as toxicity management.

Several limitations of the present study should be acknowledged. First, this is a randomized but non-comparative phase II trial and is not designed to formally compare SOX + ZOL with CapeOX + ZOL. Therefore, any apparent differences between treatment arms should be interpreted with caution. Second, the study is not powered to establish superiority or non-inferiority of SOX + ZOL relative to currently recommended regimens. Rather, the primary purpose of this trial is to generate preliminary efficacy and safety data and to inform the design of future confirmatory studies.

This trial is designed to evaluate the efficacy and safety of SOX + ZOL in patients with HER2-negative, CLDN18.2-positive unresectable or recurrent gastric cancer. By prospectively assessing this regimen with CapeOX + ZOL as a reference arm, the study will provide important information regarding the clinical activity and tolerability of SOX + ZOL in Japanese patients.

## Supplementary Information

Supplementary Material 1.

## Data Availability

The trial is ongoing and no data are available.
